# Pathogenicity of Nipah henipavirus Bangladesh in a swine host

**DOI:** 10.1038/s41598-019-40476-y

**Published:** 2019-03-26

**Authors:** S. B. Kasloff, A. Leung, B. S. Pickering, G. Smith, E. Moffat, B. Collignon, C. Embury-Hyatt, D. Kobasa, H. M. Weingartl

**Affiliations:** 1 National Centre for Foreign Animal Disease, Canadian Food Inspection Agency, Winnipeg, Manitoba Canada; 2 Present Address: National Microbiology Laboratory, Public Health Agency of Canada, Winnipeg, Manitoba Canada; 3 Department of Medical Microbiology, University of Manitoba, Winnipeg, Manitoba Canada

## Abstract

In 1998 an outbreak of fatal encephalitis among pig farm workers in Malaysia and Singapore led to the discovery of Nipah henipavirus (NiV), a novel paramyxovirus closely related to Hendra henipavirus with case fatality rates of nearly 40%. Following its initial emergence nearly annual outbreaks of NiV have occurred in Bangladesh with a different, NiV Bangladesh, genotype, where the role of pigs in its transmission remains unknown. The present study provides the first report on susceptibility of domestic pigs to NiV Bangladesh following experimental infection, characterizing acute and long-term phases of disease and pathogenesis. All pigs were successfully infected with NiV Bangladesh following oronasal inoculation, with viral shedding confirmed by a novel genotype-specific qRT-PCR in oral, nasal and rectal excretions and dissemination from the upper respiratory tract to the brain, lungs, and associated lymphatic tissues. Unlike previous NiV Malaysia findings in pigs, clinical signs were absent, viremia was undetectable throughout the study, and only low level neutralizing antibody titers were measured by 28/29 days post-NiV-B infection. Results obtained highlight the need for continued and enhanced NiV surveillance in pigs in endemic and at-risk regions, and raise questions regarding applicability of current serological assays to detect animals with previous NiV-B exposure.

## Introduction

Since its emergence in 1998, Nipah henipavirus (NiV) outbreaks in Southeast Asia have resulted in the loss of hundreds of human lives and hundreds of million dollars in lost revenue to the agricultural sector. Classified as a Risk Group 4 pathogen with demonstrated epizootic, zoonotic, and human-to-human spread, NiV is one of the prototypic species in the genus *Henipavirus*, historically considered unique amongst the paramyxoviruses for their high virulence combined with broad host range^1^. However, the recent expansion of the genus to include Cedar, Mojiang, and Ghanaan bat henipaviruses suggests that not all species possess these characteristics^2–4^.

The discovery of Nipah henipavirus (NiV) followed an initial outbreak of encephalitis in pig farm workers in Peninsular Malaysia and Singapore, where 276 cases were reported with a case fatality ratio (CFR) of nearly 40%. It was not until the epidemiological link was established that attention was given to a concurrently circulating disease in pigs, as clinical signs in affected animals were similar to those observed in other swine diseases of the region. Further, overall morbidity and mortality rates were not significantly above normal. This lapse in diagnosis resulted in the continued sale and movement of pigs from infected farms, facilitating the geographic spread of disease. The epidemic was eventually halted by a stamping out policy where nearly 1.1 million of the 2.4 million pigs in Peninsular Malaysia were culled by the summer of 1999^5,6^.

Though largely lacking clinical disease, the infection rate in pigs was estimated at 100%. Molecular characterization of NiV isolated from pigs suggested two possible introductions of the virus from its pteropid bat host over the course of the Malaysia outbreak^7^ while virus isolates from human infections showed over 99% homology with those from pigs. The critical role of pigs as intermediate hosts in the spread of the NiV-Malaysia (NiV-M) outbreak was further validated by the absence of clinical disease or evidence of exposure to NiV in persons residing in vicinity of where virus was isolated from bats^8^. No further outbreaks of NiV have been reported in Malaysia since the last detection of a seropositive pig in May of 2000; however molecular characterization of a patient isolate from a 2014 henipavirus outbreak in the Philippines interestingly suggests NiV-M as the possible aetiological agent^9^.

In 2001 the first Nipah henipavirus outbreak was reported in Bangladesh with direct transmission from bats to people, largely through consumption of contaminated date palm sap and without involvement of any amplifying host. Since then, outbreaks and isolated transmission events have been reported on a nearly annual basis in Bangladesh as well as in neighbouring West Bengal State of India^10,11^. Infections with Bangladesh genotype viruses (NiV-B) have differed in multiple ways from the original Malaysia outbreak, including significantly higher associated CFRs of 75–100%^10,12^. Further, over half of identified NiV-B cases between 2001 and 2007 resulted from person-to-person transmission, while no human-to-human transmissions were recorded in the initial 1998–1999 Malaysia outbreak^13^. Severe respiratory disease, infrequently seen in NiV-M cases, was observed in over 60% of NiV-B infections, and is likely the major factor contributing to these distinct transmission patterns. Perhaps the greatest difference between the NiV-B and NiV-M outbreaks lies in the spillover mechanism from the *Pteropus* bat reservoir to the human population. The emergence of NiV in Malaysia and Singapore followed more than one spillover event into the swine population, with pigs acting as intermediate hosts and transmitting the virus to persons working in close contact with these animals. Conversely, primary human cases with NiV in Bangladesh and bordering India have been linked to consumption of fresh or fermented date palm sap contaminated by infected bat excretions^14,15^. While epidemiological investigations have indicated certain case clusters were more likely to have come in contact with a herd of pigs, sick goat or cow^16^, no virus has been isolated from livestock in the area and serological surveys of domestic animals in Bangladesh found antibodies against NiV soluble G protein were non-neutralizing^17^. As such, the lack of documented natural outbreaks leads to the question of whether pigs are susceptible to NiV-B and what potential roles they may play as amplifying hosts in future outbreaks.

Nipah henipavirus is considered a category C agent for potential bioterrorism, and is a prioritized pathogen under the World Health Organization’s Research and Development Blueprint^18^. Accordingly, a clearer understanding of the host range of this virus combined with data on susceptibility and pathogenesis in domestic livestock is of critical importance for implementation of appropriate surveillance and detection systems. Here, we present the first experimental evidence that domestic pigs are susceptible to infection with NiV-B following oronasal inoculation.

## Materials and Methods

### Virus

Rescue of a Nipah Bangladesh henipavirus was performed using a modified pSP72 (Promega) vector-based plasmid system developed at Public Health Agency of Canada’s Special Pathogens unit^19^. The sequence used was based on the complete Genbank reference sequence (Accession #AY988601) with modifications to the fusion gene to reflect coding changes in more recently circulating isolates (Accession numbers JN808863 and JN808857); positions 7278-79 (TC to CT), resulting in amino acid S207L, and position 7414 (G to A), resulting in AA G252D. Briefly, 293 Griptite cells (Invitrogen) were transfected with plasmids encoding the Nipah henipavirus Bangladesh genome along with Nipah Bangladesh N, P and L pCAGGs helper plasmids. Transfections were performed using LT1 transfection reagent (Mirus) in OPTIMEM and transferred immediately to the BSL-4 laboratory. Supernatants from transfected cells were subsequently used to infect Vero E6 cells. Stocks of Nipah Bangladesh (Passage 2 in Vero E6) in TriPure isolation reagent were sent for sequencing analysis by the NCFAD Genomics unit to ensure no changes were acquired. Nipah henipavirus Malaysia stocks were generated from a NCFAD experimental porcine lung isolate, plaque purified and passaged (P4) in Vero 76 cells. Both viruses were titrated via TCID_50_ and standard plaque assay in Vero76 cells with a 1.75% CMC overlay and visualized with crystal violet.

### Replication Kinetics in swine cells *in vitro*

Comparative replication kinetics of NiV-B and NiV-M were assessed in swine cell lines over a period of 72 hours. ST cells, IPAMs (Immortalized porcine alveolar macrophage clone 3D4/31), and Vero76 cells seeded in 24-well plates at 130,000 cells/cm^2^, 250,000 cells/cm^2^ and 100,000 cells/cm^2^, respectively, were infected the following day with NiV-B and NiV-M in parallel at 0.01 moi. At 1 hour post-infection inoculum was removed and replaced with 1 mL DMEM 2% FBS. Supernatants from three infected wells per cell type were harvested at 1, 24, 48 and 72 hours post-infection (hpi) and viral titer was quantified by TCID_50_ assay in Vero76 cells and semi-quantitative real time RT-PCR as described below.

#### RNA Extraction

Tissue culture supernatants were inactivated with TriPure isolation reagent (Roche) at a ratio of 1:9 or Trizol LS Reagent (Invitrogen) at a ratio of 1:4 in the case of whole blood. Tubes were vortexed immediately and left at room temperature for a minimum of 20 minutes prior to packaging for removal from BSL-4 for downstream processing. Total RNA from experimental samples was extracted using the Direct-zol™ RNA MiniPrep (Zymo Research) according to manufacturer’s instructions and stored at −80 °C until use. Exogenous armored enterovirus RNA (Asuragen, Austin, TX), added to Tri reagent, was used as an extraction control.

#### qRT-PCR for detection of NiV-B and NiV-M

Genotype-specific semi-quantitative real time (q) RT-PCR was utilized to detect RNA from NiV-M and NiV-B-infected supernatants. qRT-PCR on NiV-M-infected samples was performed using primers and probe targeting the viral nucleoprotein gene as described by Guillaume *et al*. (2004). For specific detection of NiV-B, primers were designed to cover the fusion gene non-coding region (NCR) containing a 6-nucleotide insert that is absent in NiV-M in order to serve as a specific differential assay. Sequences of assay components, using numbering based on Nipah Bangladesh Accession # AY988601, are as follows:

NiV-B_6503_Forward: 5′ GTGTTAAACCTAGGAGACCCTTC 3′

NiV-B_6631_Reverse: 5′ GGTCTACCAACTGCTTTGATTTG 3′

NiV-B_Probe: /56-FAM/AGTCAGGTCGCGGGAATACAAACA/ZEN//3IaBkFQ/

Mastermix for qRT-PCR was prepared using the Invitrogen SuperScript™ III Platinum™ One-Step qRT-PCR Kit w/ROX according to manufacturer’s specifications, using 5 pmol of each NiV primer and 6 pmol of NiV-specific probe per reaction. Reaction conditions were as follows: 50 °C for 15 min, 95 °C for 2 min, and 40 cycles of 95 °C for 15 s followed by 60 °C, 30 s. Runs were performed using a Rotor-Gene Q (Qiagen, Software version 2.3.1.49) and semi-quantitative results were calculated based on a plasmid-based standard curve for NiV-B fusion gene or NiV-M nucleoprotein gene as appropriate.

### *In vivo* Studies

#### Ethics Statement

Experimental design, including housing conditions, sampling regimen, and humane endpoints, was approved by the Animal Care Committee of the Canadian Science Centre for Human and Animal Health in AUD #C-17-003 (Pathogenesis of Nipah henipavirus Bangladesh Strain in Pigs and Development of Molecular Diagnostic Tools for its Detection) and all procedures and housing conditions were in strict accordance with the Canadian Council on Animal Care guidelines. Group housing was carried out in the BSL-4 large animal cubicle, and animals were provided with commercial toys for enrichment and access to food and water ad libitum. All invasive procedures, including experimental inoculation and sample collection (nasal washes, rectal swabs, and blood collection) were performed under isoflourane gas anaesthetic, and animals were euthanized with a sodium pentobarbital solution.

#### Experimental Challenge *In vivo*

Experimental infection of Landrace pigs was carried out over two consecutive trials, with “Group 1” designated as a short-term infection (Pigs 1–6) followed by a long-term study using age-matched pigs in “Group 2” (Pigs 7–10). All animals underwent at least one week of acclimatization in a large animal cubicle at NCFAD prior to virus challenge at 5–6 weeks of age. Both experimental groups received a dose of 2.5 × 10^5^ pfu of NiV-B in a final volume of 3 mL per animal via oral (1 mL) and intranasal (1 mL per nostril) route under isoflourane gas anaesthetic. Two additional control pigs were euthanized prior to Group 1 inoculation to ensure health status of animals and provide samples for negative controls in experimental assays. Rectal temperatures and general observations of clinical signs were recorded daily.

#### Sample Collection

All samples collected throughout the experiment from swabs, washes and bleeds were immediately subdivided into multiple aliquots and frozen at −70 °C for later processing. Tissue samples obtained during post-mortem analyses were similarly subdivided, with sections dedicated for histology placed in 10% formalin, and those dedicated for qRT-PCR or virus isolation cut, weighed, and aliquoted into multiple bead mill tubes for downstream homogenate preparation.

***Group 1*** (***Short-term infection***). Nasal washes, rectal swabs (in 1 mL PBS), oral fluid from ropie chews, and blood were collected prior to inoculation and between days 1–7 post-infection from all remaining experimental animals. Euthanasia was carried out at pre-determined experimental endpoints on two animals per day at day post-infection (dpi) 2, 4 and 7. A total of 22 samples were harvested during post-mortem examination for detection of viral RNA, including: olfactory bulb, turbinate, trachea, lung, submandibular lymph node, bronchial lymph node, tonsil, meninges, trigeminal ganglion, cerebellum, cerebrospinal fluid, hindbrain, forebrain, adrenal gland, thymus, thyroid, kidney, liver, heart, pancreas, skeletal muscle and spleen.

***Group 2*** (***Long-term infection***). Nasal washes, rectal swabs, oral fluid and blood were collected at regular intervals up to 14 dpi, with alternate pigs sampled on each day to minimize stress associated with anaesthesia. Blood from all four pigs was additionally collected on day 17, 21, 24 and immediately prior to euthanasia on day 28 (pigs 7 and 8) or 29 (pigs 9 and 10) to assess seroconversion. Twelve post-mortem samples, including olfactory bulb, lung, meninges, trigeminal ganglion, cerebellum, cerebrospinal fluid, hindbrain, forebrain, and thymus, were harvested from each animal for viral RNA detection.

#### RNA Extraction, qRT-PCR, and virus isolation

Pre-weighed, frozen tissue sections in Precellys bead mill tubes were thawed and topped up with DMEM to make 10% w/v tissue homogenates. Tubes were processed using the Bertin Minilys personal tissue homogenizer and clarified at 1500 RPM. Clarified homogenates, swabs and fluids collected from experimental animals were inactivated with TriPure isolation reagent and extracted in duplicate as described in section 2.1. qRT-PCR was performed on all extracted samples using NiV-B-specific primers and probe as described in section 2.2. Selected qRT-PCR positive samples were also tested for virus isolation through standard plaque assay in Vero76 cells using freshly prepared homogenates from new aliquots of frozen tissue.

### Immunohistochemistry

Tissues fixed in 10% neutral phosphate buffered formalin were routinely processed and sectioned at 5 µm. Paraffin-embedded sections were quenched for 10 minutes in aqueous 3% hydrogen peroxide and rinsed in MilliQ water. Epitopes were retrieved using Biocare EDTA solution in a Biocare Medical Decloaking Chamber and subsequent staining was performed using a Dako autostainer. For specific detection of Nipah henipavirus, sections were incubated with the anti-NiV-Mouse monoclonal antibody F45G4^20^ at a 1:4000 dilution at room temperature for 30 minutes. Visualization was achieved using a horseradish peroxidase labelled polymer, EnvisionM+ system (anti-mouse) (Dako, USA) for 30 minutes followed by addition of chromogen diaminobenzidine (DAB). Sections were counter stained with Gill’s hematoxylin.

### *In situ* Hybridization

Tissues fixed in 10% neutral phosphate buffered formalin were routinely processed, paraffin-embedded and sectioned at 5 µm. Tissue sections were processed as per manufacturer’s instructions for RNAscope® 2.5HD Detection Reagent – Red kit using the V-Nipah-StrM.B.-N probe (Advanced Cell Diagnostics Cat No. 439251). The sections were then counter stained with Gill’s hematoxylin, dehydrated, cleared and coverslipped.

### Serum Neutralization Assays

Neutralizing antibodies titers in Group 2 sera were determined via plaque reduction neutralization test against NiV-B. Serial two-fold dilutions of heat inactivated (1 hour at 56 °C) sera were incubated for 1 hour at 37 °C with equal volumes of virus prepared at a concentration of 70 pfu/50 μl. One hundred microliters of each virus-serum mixture was then added to duplicate wells of Vero76 cells in a 48-well format, incubated for 1 hour with at 37 °C, and overlaid 500 μl of a 1.75% CMC overlay per well. Plates were then incubated at 37 °C for 48 hours, fixed with 10% buffered formalin and stained with 0.5% crystal violet. Serum dilutions resulting in >90% reduction of plaque counts compared to virus controls were considered positive for virus neutralization.

### Indirect NiV ELISA

Nunc F Flat bottom plates were coated with 100 ng per well of NiV N recombinant antigen^21^ or 20 ng per well of Nipah soluble G antigen^22^, both of NiV-M origin, in 100 µl/well 0.06 M carbonate/bicarbonate buffer (pH 9.6) overnight at 4 °C. Plates were washed 5 times with PBS 0.01 M PBS (pH 7.2)/0.05% Tween 20 (PBS-T) and blocked with a 3% BSA/10% horse serum/0.1% Tween 20/0.01 M PBS (pH 7.2) blocking buffer for 1 h with shaking at 37 °C. Test sera was added at a 1:100 dilution to duplicate wells and incubated for 1 h with shaking at 37 °C. Following five washes with PBS-T, plates were incubated for 1 hour with shaking at 37 °C with 100 μl/well peroxidase-labelled goat anti-Swine IgG antibody (KPL) diluted 1:1000 in blocking buffer. Plates were washed as above with PBS-T and reactions were developed by adding 100 μl/well of 2,20-azino-bis[3-ethylbenzothiazoline-6-sulphonic acid] (Roche Diagnostics) and read at 405 nm on a BioTek Epoch Microplate Spectrophotometer. Cut-off absorbance values for the N and soluble G ELISAs were 0.32 and 0.142, respectively.

## Results

### Comparative Replication Kinetics of NiV-B and NiV-M in porcine cells

**Figure 1:**
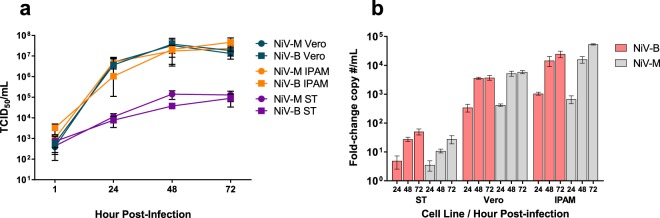
Replication Kinetics of NiV-B and NiV-M in swine cells. Porcine ST and IPAM cell lines plus Vero 76 cells were infected with NiV-B and NiV-M at moi = 0.01. Supernatants were harvested at T = 1, 24, 48 and 72 hpi for virus quantitation by (**a**) TCID_50_ assay and (**b**) genotype-specific qRT-PCR. Bars indicate mean fold change in viral copy numbers per mL compared to T = 1 hpi. Values shown are means and standard deviations of triplicate samples from one of two representative experiments.

The ability of NiV-B to infect and replicate in swine-origin cells was compared with NiV-M over a 72 hour time course. Differences in susceptibility were observed between the cell lines, with IPAMs supporting significantly higher levels of virus replication than ST cells. Overall, NiV Malaysia infection caused slightly enhanced CPE in infected cells compared to NiV Bangladesh; however the amount of infectious virus recovered from supernatants was nearly identical between the two viruses for each cell line tested (Fig. 1a, Supplementary Fig. S1). By 24 hpi, both NiV-B and NiV-M induced mild CPE in infected IPAMs, which increased in severity over the duration of the experiment. After 72 hours IPAMs infected with NiV-B showed severe CPE while NiV-M infection led to total destruction of the monolayer. Vero 76 cells, included as positive controls for virus replication, showed complete CPE by 48 hpi after infection with both viruses. For further comparison, qRT-PCR was performed on all harvested supernatants using genotype-specific assays. While the coding sequence of NiV-B is mismatched at only two nucleotides per primer and a single nucleotide of the probe in the classical NP-targeted assay, a differential genotype-specific qRT-PCR assay was designed and utilized for specific detection of NiV Bangladesh (Supplementary Fig. S2). Quantitation of viral replication by qRT-PCR (Fig. 1b) reflected CPE observations and TCID_50_ results. In ST cells, only minor, gradual increase in replication was measured by qRT-PCR and TCID_50_, however no cytopathic effect was observed at any point post-infection, irrespective of NiV genotype examined (Supplementary Fig. S1).

### Experimental Infection of Pigs with Nipah henipavirus Bangladesh (NiV-B)

Daily measurements of rectal temperatures showed no fever at any point during the course of infection (temperature range from 38.4 °C to 39.8 °C) (Supplementary Fig. S3). On day 2 pi, Group 1 pigs appeared slightly slower-moving than usual. For the remainder of the short term experiment, and throughout the duration of the long-term Group 2 study, pigs appeared normal, bright and alert, and respiratory and neurological signs were absent.

#### Virus shedding

**Figure 2:**
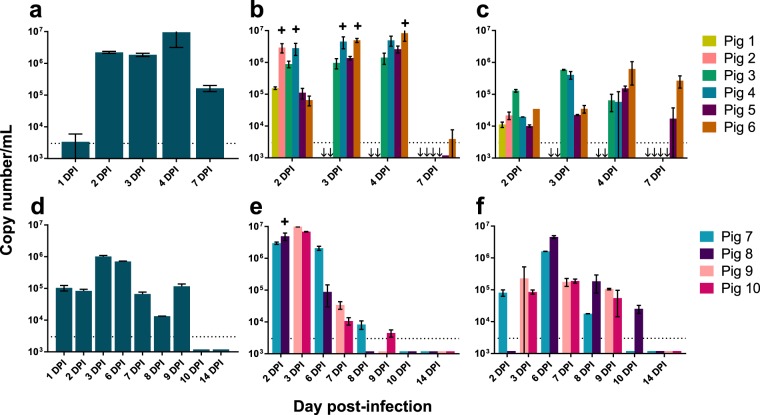
Shedding of NiV Bangladesh in infected pigs. qRT-PCR results for detection of NiV-B in oral fluid (**a**,**d**), nasal washes (**b**,**e**) and rectal swabs (**c**,**f**) collected throughout the course of infection from pigs in experimental Group 1 (**a-c**) and Group 2 (**d**–**f**) are shown. Plus signs (+) denote samples positive for virus isolation, arrows (→) indicate missing values due to preceding experimental endpoints, dotted lines indicate limits of detection and flat line bars denote negative results or those below limits of detection.

To assess virus shedding, a number of samples were collected at regular intervals for the entirety of the Group 1 experiment and up to 14 dpi for Group 2. All of the Nipah Bangladesh-infected pigs in both experimental groups had viral RNA in nasal and rectal excretions, and oral shedding was detected on a group basis. NiV-B RNA was present above the detection limits in oral fluids by qRT-PCR between DPI 2 and 7 for Group 1 animals (Fig. 2a) and on all sampling days up to 9 dpi in pigs from Group 2 (Fig. 2d). Peak nasal wash titers were observed between dpi 2 and 4 in both experimental groups, with positive virus isolations complementing qRT-PCR results on a selection of samples from dpi 2,3 and 4 (Fig. 2b,e). The prolonged sampling period for Group 2 pigs revealed low-level shedding in nasal washes up to day 9 post-infection (Fig. 2e). Viral RNA was detectable in rectal swabs beginning at 2 dpi, exceeded nasal wash titers by 6 dpi and persisted though day 10 post-infection (Fig. 2c,f).

#### Tissue Tropism

**Figure 3:**
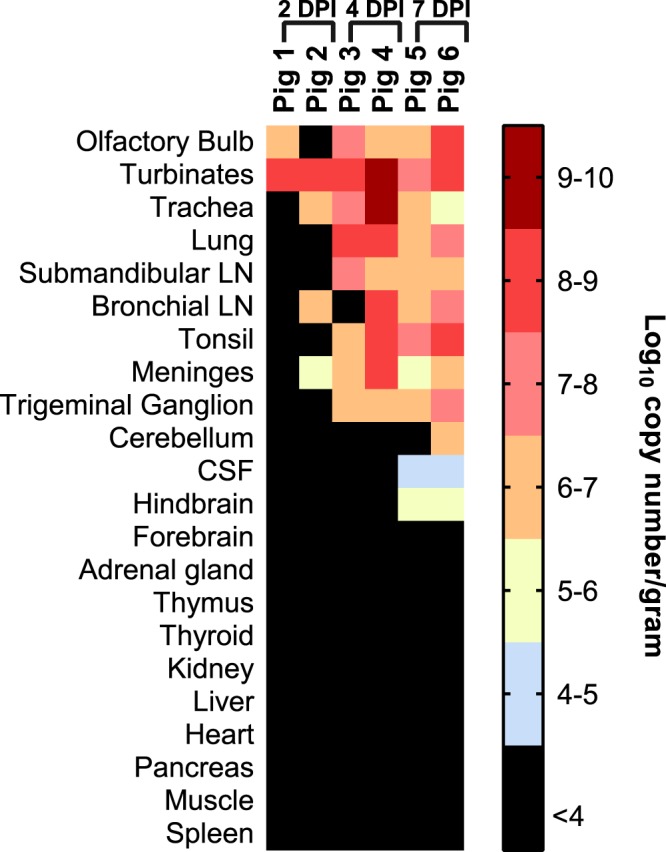
Detection of NiV-B in twenty-two post-mortem samples. Semi-quantitative qRT-PCR results targeting the non-coding region of NiV-B fusion gene from samples harvested during necropsies at Group 1 experimental endpoints. Samples indicated in black were either negative or below cut-off value.

For an understanding of disease progression, sequential necropsies were performed on days 2 (Pigs 1 & 2), 4 (Pigs 3 & 4) and 7 (Pigs 5 & 6) post-infection. A total of twenty two post-mortem samples were collected from each animal, including tissues from the respiratory, nervous, and lymphatic systems plus other visceral organs. On day 2 post-infection, viral RNA was detected in nasal turbinates (2/2 pigs), trachea (1/2), bronchial lymph node (1/2), olfactory bulb (1/2), and meninges (1/2), of infected animals (Fig. 3). Results from virus isolation, performed using freshly prepared homogenates from frozen tissue sections, also confirmed the presence of infectious virus in homogenates of nasal turbinates from both pigs necropsied at dpi 2 (Table 1).

**Table 1:** Virus isolation results from post-mortem samples.

Sample	DPI 2				DPI 7	
	Pig 1	Pig 2	Pig 3	Pig 4	Pig 5	Pig 6
Olfactory bulb	+	/^a^	/	−	−	−
Turbinates	+	+	+	+	−	−
Trachea	/	+	−	+	−	−
Lung	/	/	+	+	−	−
Submandibular LN	/	/	+	+	−	−
Bronchial LN	/	−	/	−	−	−
Tonsil	/	/	+	+	/	+
Meninges	/	−	−	+	/	−
Trigeminal ganglion	/	/	−	−	−	−
Cerebellum	/	/	/	/	/	−
CSF	/	/	/	/	−	−

^a/^Not attempted.

**Figure 4:**
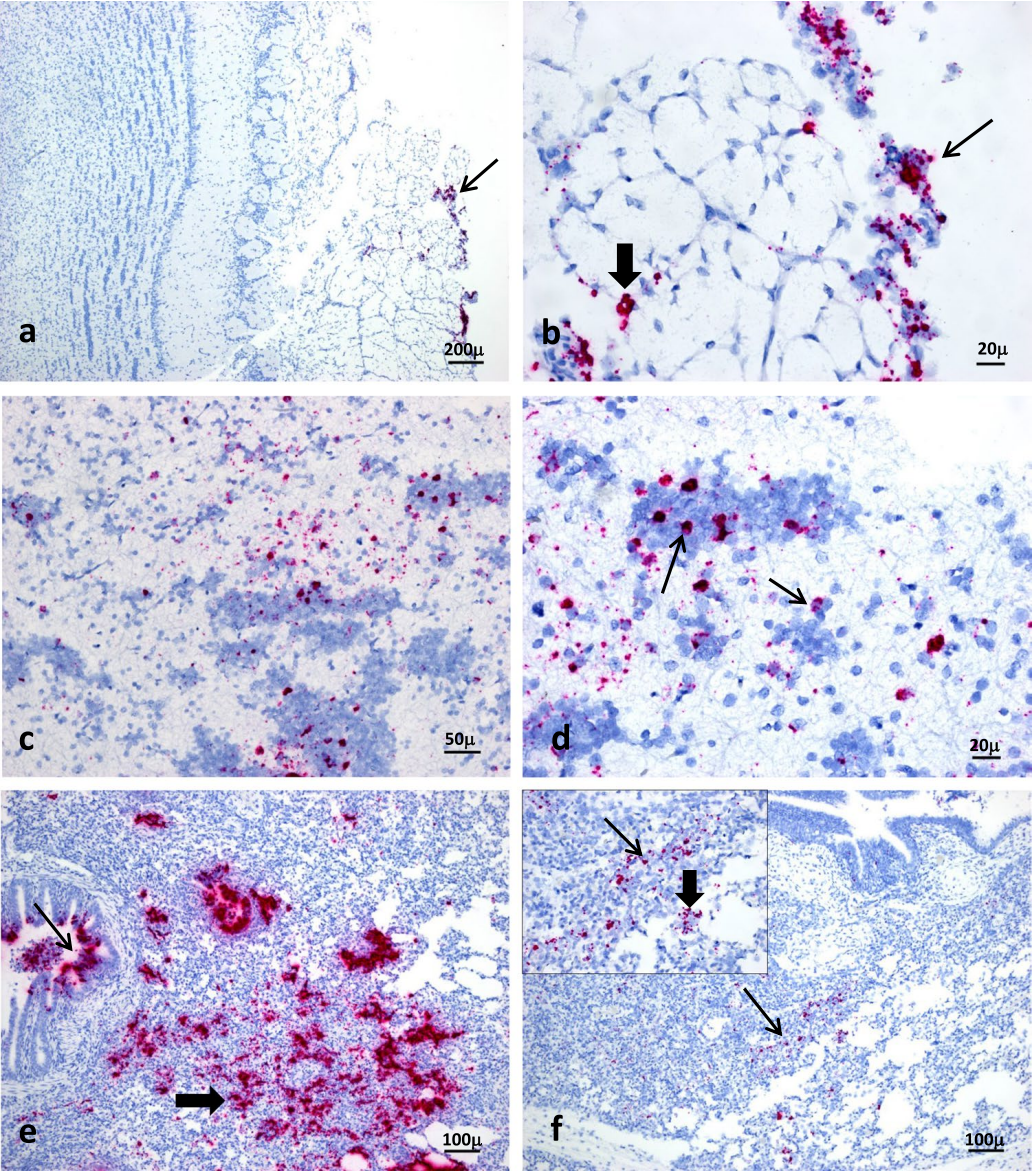
Detection of NiV-B RNA in olfactory bulb and lung by ISH at 4 and 7 days post-oronasal inoculation. At 4 dpi multifocal areas of intense viral RNA signal were observed in the olfactory nerve layer (arrow) (**a**). Higher magnification of (**a**) shows viral RNA in meningeal cells (thin arrow) and supporting cells of the nerve fibre layer (thick arrow) (**b**). By 7 dpi foci of positive staining found within the granular cell layer (**c**), particularly in interneurons (arrows) (**d**). Infected lungs show abundant viral RNA signals throughout sections in both bronchioles (thin arrow) and interstitium (thick arrow) at 4 dpi (**e**), but limited to few foci throughout the lung interstitium (arrow) by 7 dpi. Inset: Staining observed in cells within alveolar walls (thin arrow) as well as in cells that appear morphologically to be macrophages (thick arrow) (**f**).

By 4 dpi all respiratory tissues, including lungs, were positive for both viral RNA (all samples) and virus isolation (at least 1 pig per tissue type). NiV-B titers detected on this day reached their peak levels in the above-mentioned respiratory tissues as well as meninges, submandibular lymph nodes, and bronchial lymph nodes. Tonsils and trigeminal ganglia of both animals examined on day 4 post-infection were also positive by qRT-PCR and/or virus isolation (tonsils) (Fig. 3, Table 1). *In situ* hybridization (ISH) analyses of olfactory bulbs and lungs collected at 4 dpi confirmed the above results, revealing viral RNA in the meningeal cells and supporting cells of the nerve fiber layer surrounding the olfactory bulb, and extensive staining in both bronchioles and the interstitium of lung sections (Fig. 4). ISH results were confirmed by immunohistochemistry (Supplementary Fig. S4).

After seven days of infection, viral RNA persisted in samples from the brain, including hindbrain (2/2 pigs), CSF (2/2) and the cerebellum from one of two animals. Analyses of tissue sections by ISH revealed viral RNA persisting in a few foci corresponding to interneurons within the granular layer of the olfactory bulb. Within the lungs, viral RNA was no longer present in the bronchioles by 7 dpi and instead was detectable in only a few foci throughout the lung interstitium, localized to cells within the alveolar walls as well as cells with morphological appearances of alveolar macrophages (Fig. 4). Other previously positive tissues continued to harbor viral RNA though infectious virus was only recovered from the tonsil of a single animal (Pig 6) at this stage of infection (Fig. 3, Table 1). Group 2 animals, euthanized at 28 and 29 days post-infection, had no detectable viral RNA in any of the 11 post-mortem samples collected. No viremia was detected by qRT-PCR in either group of pigs during the acute phase of infection in whole blood, serum or peripheral blood mononuclear cells (results not shown).

#### Neutralizing Antibody Response

**Figure 5:**
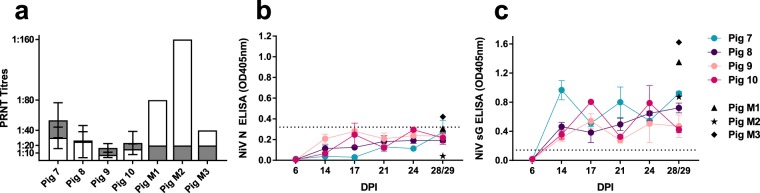
Antibody response following NiV-B infection. (**a**) Neutralizing antibody titers conferring >90% plaque reduction of NiV-B (grey bars) or NiV-M (white bars) in 28/29 dpi sera from Group 2 pigs. (**b**) Antibody responses measured by indirect ELISA against NiV N antigen (cutoff = 0.32) or (**c**) NiV soluble G antigen (cutoff = 0.142). Results are shown for individual animals and indicate mean plus standard deviation of two independent experiments. Archived sera from past NiV-M experiments (Pig M1-M3) were included as positive controls for ELISA and cross-neutralization assays.

Development of serum antibody responses in NiV Bangladesh-infected pigs was assessed through plaque reduction neutralization tests (PRNT) and indirect NiV N and NiV soluble G protein-based ELISA (Fig. 5). Sera harvested from Group 2 pigs showed only modest anti-NiV-B activity at 4 weeks post-infection, with neutralizing titers (resulting in >90% plaque count reduction) ranging from 1:20 to <1:80. When assessed for cross-neutralizing activity using heterologous NiV-M virus, titers of Group 2 sera were approximately 50% lower than those observed with NiV-B virus. Similarly, neutralization of NiV-B by heterologous sera from past experimental NiV-M infections showed a low degree of cross-neutralization compared to titers observed for homologous virus (Fig. 5a). When tested with Hendra virus, even a 1:10 dilution of sera from NiV-B-infected pigs showed no detectable cross-neutralizing activity (results not shown). This observation reflected previous findings, where pigs vaccinated with adjuvanted recombinant soluble Hendra virus G protein elicited markedly lower neutralizing antibody titers to NiV compared to Hendra, and were not protected from NiV challenge^23^.

Antibody responses from sera harvested throughout the Group 2 pig experiment were also measured by indirect ELISA against NiV N and NiV soluble G antigens. Results from the NiV N ELISA showed generally low level antibody activity starting at 14 dpi through to 28/29 dpi, with mild fluctuations observed over the experimental course and no direct correlation with plaque reduction activity (cut off = 0.32) (Fig. 5b). With soluble G antigen, indirect ELISA results (cut off = 0.142) showed similar patterns of fluctuation between dpi 14 and 28/29. Here, OD_405nm_ readings had greater correlation with virus neutralization results, with the highest anti-soluble G activity in Pig 7 and Pig 8 final sera (Fig. 5c) reflecting the associated PRNT results.

## Discussion

The Bangladesh genotype of Nipah henipavirus has caused repeated outbreaks of human disease in Bangladesh and nearby India since the year 2000. Unlike the initial NiV-M epidemic in Malaysia and Singapore, with all human cases attributed to transmission of virus through close contact with infected pigs, outbreaks in Bangladesh have been largely linked to direct virus transmission from bats to humans through consumption of NiV-B-contaminated food products. Suggestive serological data combined with the lack of documented NiV infection in pigs from Bangladesh has raised the question as to whether differences between the two genotypes cause a decreased susceptibility to pigs with the Bangladesh strain. The present work contains the first evidence that domestic pigs are susceptible to Nipah Bangladesh following oronasal infection, with spread to respiratory, nervous and lymphatic tissues and shedding in oral and nasal secretions and excreta. Interestingly, signs of clinical disease were absent in all infected animals.

Experimental oronasal inoculation of 5–6 week old piglets with NiV-B led to productive infection with viral dissemination through the respiratory tract and nervous system, and detectable shedding up to 9 dpi in both nasal wash and oral fluid. Tissue invasion in NiV-B-infected pigs followed similar patterns to those reported in experimental swine infections with NiV-M. qRT-PCR of tissues collected during necropsy were positive for Nipah henipavirus RNA as early as 2 dpi in respiratory, nervous and lymphoid tissues. By 4 dpi lung involvement was detected, as was infection of further nervous and lymphatic tissues. However, a number of noteworthy differences were observed between the present NiV-B swine experiments and previous experimental findings with NiV-M.

Pigs infected with NiV-B had detectable virus in rectal swabs via qRT-PCR from the first sampling point at 2 dpi through the experimental endpoint for Group 1 (7 dpi), and up to 10 dpi in the long-term experiment. Immunohistochemical examination of formalin-fixed tissue from a naturally infected piglet during the original NiV-Malaysia outbreak revealed viral antigen in the stomach and small intestine, localized to endothelial and smooth muscle cells surrounding small arteries^24^. However, experimental infection of piglets with the same virus has yet to result in qRT-PCR positive rectal swabs, and no NiV has been detected in feces of infected pigs^25–27^. This also differs from results observed in nonhuman primate studies, where NiV-B was detected in rectal swabs only at 7 dpi while NiV-M was found starting at dpi 3^28^. Collection of intestinal tissue sections is warranted in future NiV-B experimental infections to elucidate the cellular tropism of NiV-B for these tissues in pigs. Additionally, as faeces from experimentally infected ferrets were shown to harbor NiV-B RNA^29^, testing of fecal samples from NiV-B-infected pigs should also be carried out to determine their utility as environmental diagnostic samples during a suspected outbreak.

Another major difference was found in viral tropism for the spleen. Experimental infection of pigs with NiV Malaysia has repeatedly demonstrated the presence of viral RNA in the spleen of infected animals as early as 4 dpi, albeit in low titers^23,26,27,30^. This finding was also seen in fatal human cases; with immunohistochemistry of tissues from autopsies during the Nipah Malaysia outbreak also confirming presence of viral antigen in macrophages and multinucleated giant cells in the spleen^31^. With NiV Bangladesh, viral RNA has similarly been detected in the spleens of experimentally infected hamsters, non-human primates and ferrets^28,29,32^. In the present study, however, no viral RNA was detected in the spleen of pigs infected with NiV-B at any of the experimental endpoints, and histological examination of spleen sections revealed no evident pathology. Interestingly, Stachowiak *et al*. demonstrated a significant drop in CD4+ CD8− T helper cells following *in vitro* PBMC infection with NiV-M, which would influence the development of a humoral immune response^33^. A comparative study of PBMC population dynamics following NiV-M and NiV-B infection would be extremely interesting to complement the low-level antibody responses observed in Group 2 pigs compared to previous NiV-M experimental swine infections^23,25,26,34^.

NiV Bangladesh was detected in the CNS as early as 2 days post-infection, a timeline comparable with NiV-M invasion of CNS, where the virus reaches the brain both directly through cranial nerves and via viremia and/or lymphatic circulation^30^. With NiV Bangladesh, however, the mode of invasion appears to be somewhat different, as ISH did not reveal virus in axons in the glomerular layer of the olfactory bulbs, which were qRT-PCR-positive at all experimental endpoints. As such, this suggests that NiV-B invasion of the brain did not occur directly through cranial nerves, but rather by movement of infected monocytes or lymphocytes through the blood and/or lymphatic vessels. Systemic dissemination through viremia was also noted with measles virus, also of the Paramyxoviridae family, when aerosolized infection of non-human primates led to early productive infection of cells appearing to be alveolar macrophages and dendritic cells in the lung, which then spread the virus to extra-pulmonary sites^35^. The lack of detectable viremia in serum or PBMCs by qRT-PCR combined with detection of viral RNA in the meninges and lymph nodes at early days post-inoculation suggests that invasion of the CNS by NiV Bangladesh may occur via the lymphatic system. However, crossing the blood-brain barrier cannot be excluded as the possibility remains that infection of monocytes and/or lymphocytes even below limits of detection in the blood was sufficient for viral transport to the CNS.

Clinical signs observed during the NiV swine outbreak in Malaysia included trembling, rear leg weakness, uncoordinated gait, breathing difficulties, cough and head pressing^5^. Response to infection was also somewhat age-dependent, as younger piglets presented with more respiratory signs while boars and sows more frequently displayed neurological signs or died suddenly without obvious illness. However, despite the estimated natural infection rates of 100% on infected farms, many animals showed no significant clinical signs. In experimental pig infections with NiV-M these signs have been repeatedly replicated, though often present in only a limited number of infected animals per study^25,27,34^. In the present study, using the same dose and route for experimental inoculation of pigs as used with NiV-M, no clinical signs were observed over the course of infection. However, isolation of infectious virus from nasal washes suggests that asymptomatic NiV-B-infected pigs would spread the virus to susceptible pen mates.

The delayed identification of Nipah henipavirus as the aetiological agent causing a novel disease syndrome in Malaysian pigs came at a price of lost human lives and total devastation to the swine industry in the area. While epidemiological links have been made between isolated transmission events and close contact with sick animals, no laboratory confirmed cases of NiV in livestock have been documented in Bangladesh, and outbreaks of human disease have been largely linked to direct transmission from bats through consumption of contaminated date palm sap-derived beverages^14,15^. A comprehensive serological survey of livestock in Bangladesh found that 44% of pigs included in the study were seropositive for NiV by Luminex assay, though the fact that antibodies were non-neutralizing led to the conclusion that exposure was likely to an as-yet uncharacterized virus antigenically similar to Nipah henipavirus^17^. Sera collected from pigs experimentally infected with NiV-B in the present study showed markedly lower neutralizing activity against homologous virus than titers observed with NiV-M in previous swine challenge studies^23,34^. Cross-neutralization of NiV-M was similarly low, and no neutralizing activity was achieved against Henda henipavirus. The lack of cross-neutralization between the two genotypes in pigs contrasts observations in ferrets, where a recombinant vesicular stomatitis virus expressing the surface glycoproteins of NiV-B provided complete protection from lethal challenge with heterologous NiV-M^36^. Unlike other animal models of henipavirus infection, pigs appear unique in their responses to active immunization strategies. This is best exemplified by the inability of an adjuvanted recombinant soluble Hedra virus G glycoprotein vaccine to protect pigs against challenge with heterologous NiV^23^, despite its documented ability to elicit potent cross-protection in mice, rabbits, cats, ferrets, monkeys and horses^37^.

To further characterize the porcine antibody response to NiV-B infection, sera were also tested on two ELISA platforms specific to the nucleoprotein and G glycoprotein of NiV-Malaysia. When tested with the N ELISA, these sera produced OD readings near background levels^21^. A study of the antigenic domains of NiV-M nucleoprotein pointed to three putative antigenic motifs, with the core sequence of NXTQ in motif 3 (amino acids 503–509) constituting the most antigenic region^38^. Viruses of the NiV-B genotype show significant variation in this third motif, which may also partially explain these results. Similarly, while NiV-B and NiV-M show 96% amino acid sequence homology in their attachment glycoproteins, the differences may contribute to the results observed with the soluble G-based ELISA. As space limitations prevented prolonged housing of animals, changes in antibody levels beyond the experimental endpoints could not be assessed. However, information on the duration of the antibody response and the relatively low neutralizing activity observed in sera from experimentally infected pigs would be very important for future serological surveys. Based on presented data, it cannot be excluded that animals previously infected with NiV-Bangladesh could be missed by serological-based assays.

Large animal studies in the BSL-4 laboratory provide a unique opportunity to characterize pathogenesis of emerging diseases with potentially grave impacts on the livestock industry, public health and the economy alike. An inherent limitation of such studies is the use of only a small number of animals, precluding arrival at an N of statistical significance. In the present exploratory study, the inclusion of two animals at each experimental endpoint allowed for baseline establishment of NiV-B tissue tropism and disease progression in experimentally-infected piglets; information of significant potential value for post-mortem sample collection in a hypothetical outbreak situation. Future hypothesis-driven studies with similar sampling schema would be valuable to confirm and expand on the results described herein. An additional limitation of this study was the availability of a virus isolate most closely representing the agent likely to cause a potential infection in the field. The present work was carried out with a recently described rescued NiV-B^19^ that displays a comparable pathogenicity profile to its wild type counterpart in the Syrian hamster model^32^. As NiV-Bangladesh has yet to cause documented infection of domestic pigs in natural transmission events, a field isolate from said host could not be used as inoculum. Based on future availability, comparative studies employing a minimally-passaged virus isolated from bat urine or saliva would be of significant interest given the ecology of the virus and putative exposure scenarios.

The geographic distribution of the *Pteropus* bats over Eastern Africa, Asia, Australia, and the Pacific islands^39^, combined with recent findings of henipavirus in non-pteropid hosts in China^4^ and West Africa^3^ suggests a broad area at potential risk for henipavirus disease. This combined with the movement of infected pigs and/or contaminated pig products, in addition to the deliberate release of the virus anywhere in the global swine industry, leaves a very real possibility of a NiV outbreak without respect for borders or climactic conditions. Results from the present work highlight the need for continued and enhanced surveillance in pigs in Bangladesh and other endemic regions, and potential problematics associated with the use of NiV-M based serological tests to detect NiV-B exposure. Future experiments on transmission and characterization of cellular and adaptive immune responses will be required to elucidate the dynamics of Nipah Bangladesh in pigs and provide insight on the lack of recorded natural infections in areas with high likelihood of exposure.

## Supplementary information

Supplementary Information

## Data Availability

Data and associated protocols, as well as study-related materials of a non-infectious nature can be made available to readers without undue qualifications in material transfer agreements.
