# Supplementary material for: Pathogenicity of Nipah henipavirus Bangladesh in a swine host
Source: Sci Rep. 2019 Mar 26;9:5230. doi: 10.1038/s41598-019-40476-y (PMC6435791; doi:10.1038/s41598-019-40476-y)
Supplement: Supplementary file 1 — Supplementary Information [file 41598_2019_40476_MOESM1_ESM.pdf]

Pathogenicity of Nipah henipavirus Bangladesh in a swine host

Kasloff SB*[1,2#], Leung A[2], Pickering BS[1,3], Smith G[1], Moffat E[1], Collignon B[1], Embury-Hyatt C[1], Kobasa D[2,3], Weingartl HM*[1,3]

***Corresponding Authors**:

Samantha B. Kasloff (samantha.kasloff@canada.ca)

Hana M. Weingartl (hana.weingartl@canada.ca)

Affiliations

1. National Centre for Foreign Animal Disease, Canadian Food Inspection Agency, Winnipeg, Manitoba, Canada

Samantha B. Kasloff, Bradley S Pickering, Greg Smith, Estella Moffat, Brad Collignon, Carissa Embury-Hyatt & Hana M. Weingartl

2. National Microbiology Laboratory, Public Health Agency of Canada, Winnipeg, Manitoba, Canada

Samantha B. Kasloff (#current affiliation), Anders Leung & Darwyn Kobasa

3. Department of Medical Microbiology, University of Manitoba, Winnipeg, Manitoba, Canada

Bradley S. Pickering, Darwyn Kobasa & Hana M. Weingartl

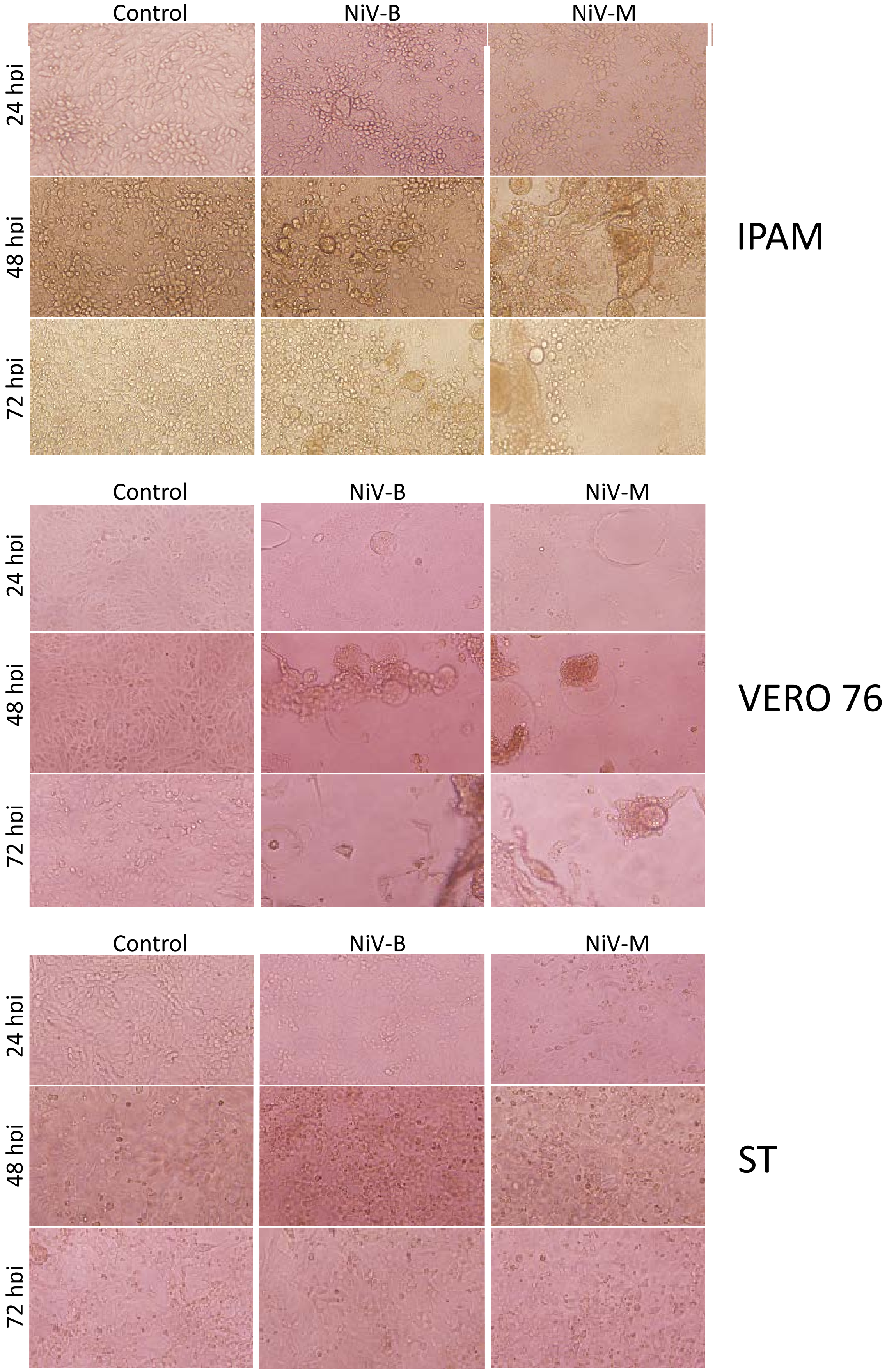

Supplementary Figure S1 - Cytopathic effect (CPE) induced by NiV-B and NiV-M in porcine cells infected at MOI=0.01.

Fusion-targeted qRT-PCR

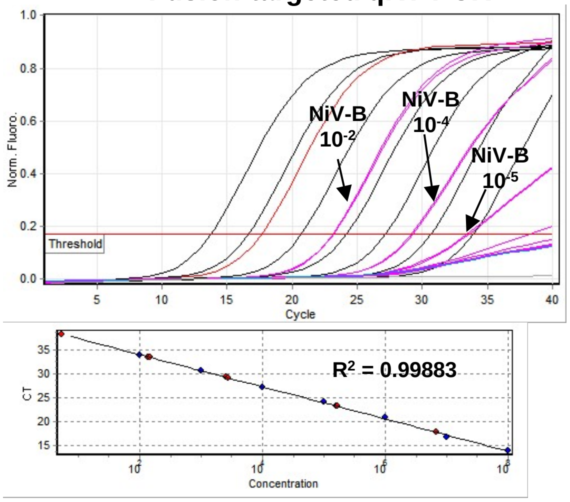

NP-targeted qRT-PCR

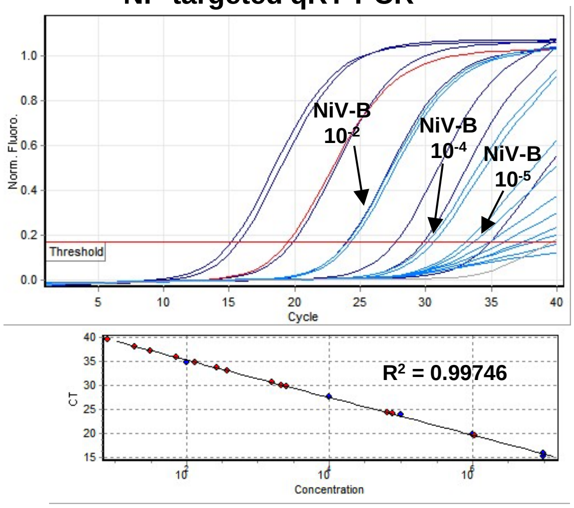

No.	Color	Name	Type	Ct	Given Conc (Copies/rxn)	Calc Conc (Copies/rxn)
1		Fusion Std 2	Standard	33.92	4.82E+01	5.28E+01
2		Fusion Std 3	Standard	30.69	4.82E+02	4.74E+02
3		Fusion Std 4	Standard	27.21	4.82E+03	5.04E+03
4		Fusion Std 5	Standard	24.20	4.82E+04	3.88E+04
5		Fusion Std 6	Standard	20.72	4.82E+05	4.14E+05
6		Fusion Std 7	Standard	16.66	4.82E+06	6.52E+06
7		Fusion Std 8	Standard	13.79	4.82E+07	4.56E+07
8		NiV-B Stock	Unknown	17.68		3.26E+06
9		NiV-B 10e-2	Unknown	23.22		7.59E+04
10		NiV-B 10e-2	Unknown	23.15		7.95E+04
11		NiV-B 10e-4	Unknown	29.18		1.32E+03
12		NiV-B 10e-4	Unknown	29.30		1.22E+03
13		NiV-B 10e-5	Unknown	33.63		6.46E+01
14		NiV-B 10e-5	Unknown	33.47		7.19E+01
15		NiV-B 10e-6	Unknown			0.00E+00
16		NiV-B 10e-6	Unknown	38.37		2.58E+00
17		NiV-B 10e-7	Unknown			
18		NiV-B 10e-7	Unknown			
19		NiV-B 10e-8	Unknown			
20		NiV-B 10e-8	Unknown			
41		H2O FUSION	NTC			

No.	Color	Name	Type	Ct	Given Conc (copies/rxn)	Calc Conc (copies/rxn)
21		NP Standard 2	Standard	34.90	1.000E+02	1.241E+02
22		NP Standard 3	Unknown	29.83		2.458E+03
23		NP Standard 4	Standard	27.70	1.000E+04	8.650E+03
24		NP Standard 5	Standard	23.99	1.000E+05	7.707E+04
25		NP Standard 6	Standard	19.86	1.000E+06	8.821E+05
26		NP Standard 7	Standard	15.19	1.000E+07	1.378E+07
27		NP Standard 7	Standard	15.75	1.000E+07	9.939E+06
28		NiV-B Stock	Unknown	19.49		1.093E+06
29		NiV-B 10e-2	Unknown	24.04		7.502E+04
30		NiV-B 10e-2	Unknown	24.31		6.399E+04
31		NiV-B 10e-4	Unknown	30.60		1.569E+03
32		NiV-B 10e-4	Unknown	30.12		2.083E+03
33		NiV-B 10e-5	Unknown	33.03		3.742E+02
34		NiV-B 10e-5	Unknown	33.63		2.623E+02
35		NiV-B 10e-6	Unknown	35.85		7.099E+01
36		NiV-B 10e-6	Unknown	38.10		1.884E+01
37		NiV-B 10e-7	Unknown			
38		NiV-B 10e-7	Unknown	37.26		3.092E+01
39		NiV-B 10e-8	Unknown	34.81		1.306E+02
40		NiV-B 10e-8	Unknown			
42		H2O NP	NTC	39.59		7.795E+00

Supplementary Figure S2. Detection of Nipah henipavirus Bangladesh RNA by real-time RT-PCR. Serial dilutions of Nipah Bangladesh virus stocks were amplified in side-by-side reactions using the classic N-targeted assay and the newly developed Fusion-specific assay.

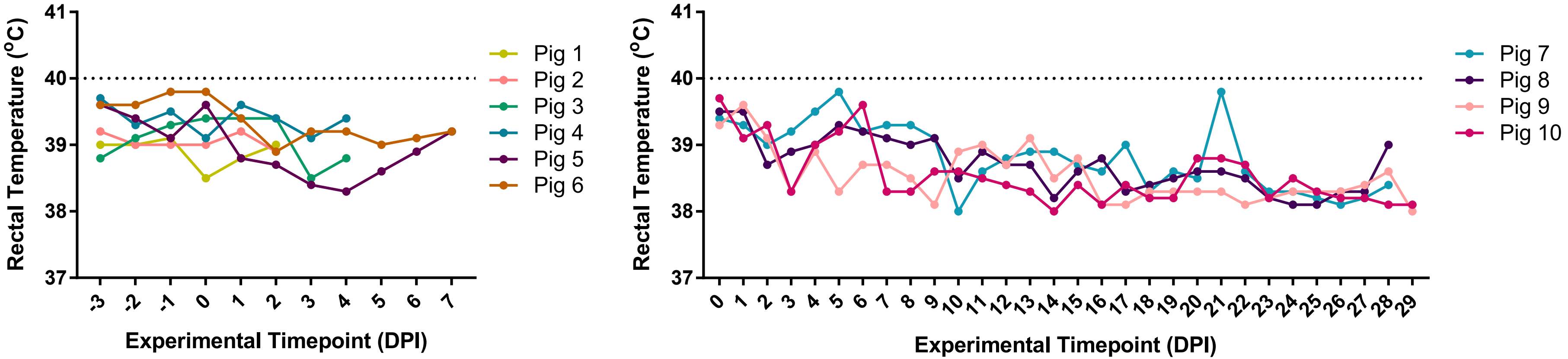

Supplementary Figure S3. Temperature monitoring of NiV-B-infected pigs. No fever was recorded at any point during the course of experiments.

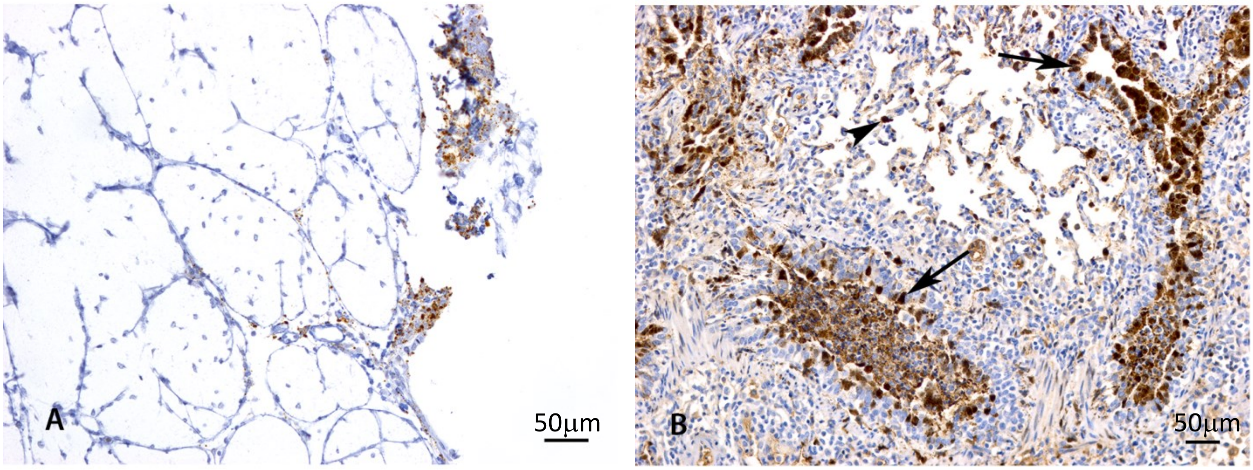

Supplementary Figure S4. Immunohistochemistry staining for detection of Nipah virus antigen in tissues collected at 4 dpi. A. Olfactory bulb B. Lung showing immunoreactivity within bronchiolar epithelial cells (arrows) and cells within the alveolar walls (arrowhead). Bar= 50 μm.
